# Association Between Toll‐Like Receptor 4 (*TLR4*) and Triggering Receptor Expressed on Myeloid Cells 2 (*TREM2*) Genetic Variants and Clinical Progression of Huntington's Disease

**DOI:** 10.1002/mds.27911

**Published:** 2019-11-14

**Authors:** Romina Vuono, Antonina Kouli, Emilie M. Legault, Lauriane Chagnon, Kieren S. Allinson, Alberto La Spada, REGISTRY Investigators of the European Huntington's Disease Network, Ida Biunno, Roger A. Barker, Janelle Drouin‐Ouellet

**Affiliations:** ^1^ John van Geest Centre for Brain Repair & Department of Neurology, Department of Clinical Neurosciences University of Cambridge Cambridge United Kingdom; ^2^ Medway School of Pharmacy University of Kent at Medway Kent United Kingdom; ^3^ Faculty of Pharmacy University of Montreal Montreal Quebec Canada; ^4^ Department of Pathology Cambridge University Hospitals NHS (National Health Service) Foundation Trust Cambridge United Kingdom; ^5^ Integrated Systems Engineering Milano Italy; ^6^ Institute for Genetic and Biomedical Research ‐ CNR Milano Italy

**Keywords:** cognitive decline, Huntington, inflammation, motor symptoms, TLR4, TREM2

## Abstract

**Background:**

Although Huntington's disease (HD) is caused by a single dominant gene, it is clear that there are genetic modifiers that may influence the age of onset and disease progression.

**Objectives:**

We sought to investigate whether new inflammation‐related genetic variants may contribute to the onset and progression of HD.

**Methods:**

We first used postmortem brain material from patients at different stages of HD to look at the protein expression of toll‐like receptor 4 (TLR4) and triggering receptor expressed on myeloid cells 2 (TREM2). We then genotyped the *TREM2* R47H gene variant and 3 *TLR4* single nucleotide polymorphisms in a large cohort of HD patients from the European Huntington's Disease Network REGISTRY.

**Results:**

We found an increase in the number of cells expressing TREM2 and TLR4 in postmortem brain samples from patients dying with HD. We also found that the *TREM2* R47H gene variant was associated with changes in cognitive decline in the large cohort of HD patients, whereas 2 of 3 *TLR4* single nucleotide polymorphisms assessed were associated with changes in motor progression in this same group.

**Conclusions:**

These findings identify *TREM2* and *TLR4* as potential genetic modifiers for HD and suggest that inflammation influences disease progression in this condition. © 2019 International Parkinson and Movement Disorder Society

Huntington's disease (HD) is an autosomal dominant neurodegenerative disorder caused by a CAG trinucleotide expansion in exon 1 of the *Huntingtin* (*HTT*) gene,^1^ which presents with a combination of motor, cognitive, and psychiatric deficits. Despite its clear genetic basis, HD patients show variable ages of onset (AoO) and progression rate, and although CAG repeat length has been shown to correlate with the AoO of motor signs,^2^, ^3^, ^4^, ^5^, ^6^, ^7^, ^8^, ^9^, ^10^ this has been of limited clinical use in predicting AoO for an individual.^11^ Moreover, patients with similar initial clinical presentations can follow very different clinical courses,^12^ with variable rates of disease progression that are poorly correlated to CAG repeat length. Hence, the CAG repeat size alone is not sufficient to reliably predict disease onset and progression, and thus there is a need to better define what other factors impact on these 2 aspects of HD.^13^, ^14^

Previously, we have shown that tau has such an influence,^15^ whereas others have reported on a number of other genetic factors that impact on these features of HD. For instance, an abnormal, but relatively short, CAG expansion leading to HD, with a relatively long CAG track in the wild‐type allele has been shown to correlate with more severe clinical features and pathology.^16^ Genetic polymorphisms adjacent to the CAG repeats have also been shown to influence disease onset^3^, ^4^, ^10^, ^17^, ^18^ as have genes related to DNA repair.^19^, ^20^, ^21^

In addition, inflammation has now been shown to be important in many chronic neurodegenerative disorders of the brain such as Alzheimer's disease (AD)^22^ and Parkinson's disease^23^ as well as HD.^24^ The evidence for a key role of inflammation to HD comes from studies looking at microglial activation on imaging and pathologically^25^ as well as peripheral cytokine profiles,^26^ which can be found early on in the disease. Inflammation‐related genetic modifiers have also been shown to influence the risk of developing neurodegenerative disorders such as sporadic AD, and this includes the triggering receptor expressed on myeloid cells 2 (*TREM2*)^27^, ^28^, ^29^, ^30^, ^31^, ^32^, ^33^ and toll‐like receptor 4 (*TLR4*).^34^, ^35^, ^36^ We therefore sought to investigate this in HD using both postmortem studies and clinical data from a large cohort of patients. Specifically, we took advantage of tissue microarrays (TMAs) to assess the expression of these proteins in the striatum^37^ and then looked at *TREM2* and *TLR4* genetic variants/single nucleotide polymorphisms (SNPs) as genetic modifiers of disease progression in a large cohort of HD patients (N = 830) obtained from the European Huntington's Disease Network (EHDN).

## Methods

### Ethics Statement

The study was approved by the local research ethics committee and the other sites of the EHDN REGISTRY project.^38^ The participants and/or the next of kin gave informed written consent for the use of genetic material and brain tissue for research according to International Conference on Harmonisation ‐ Good Clinical Practice (ICH‐GCP) guidelines (http://www.ich.org/LOB/media/MEDIA482.pdf) and the Declaration of Helsinki.

### Subjects

Human genetic material, clinical information, and CAG repeat length data were obtained from the EHDN REGISTRY^38^ (http://www.euro-hd.net/html/registry). In total, data were available from 830 patients who had a clinical and genetically confirmed diagnosis of HD (Table 1). AoO was defined as the age at which their first HD features appeared as judged by a trained neurologist either from the neurological examination or (more frequently) from the patient history as recorded in REGISTRY. Motor, functional, and cognitive features were scored at visits approximately a year apart using the Unified Huntington Disease Rating Scale (UHDRS'99).^39^ Cognitive assessments included tests of verbal fluency as well as the digit‐symbol modality and Stroop tests (word, color, and interference subtests), all of which are known to be sensitive to the disease process in HD.^40^

**Table 1:** Demographic, Genotypic, and Clinical Characteristics of the European Huntington's Disease Network Huntington's Disease Cohort

N	830
Gender, M:F	413:417
Age^a^	50.98 (12.03)
CAG repeat length of the expanded allele	44.27 (4.25)
Years since disease onset^a^	2.10 (0.93)
UHDRS motor score^b^	32.90 (20.34)
UHDRS functional score^b^	8.42 (3.56)
Cognitive score^b^	157.62 (72.77)

Group means are shown with standard deviations in parentheses.

a At enrollment.

b At first visit. Annual change in cognitive performance was assessed based on a composite cognitive score, a sum of individual scores in the verbal fluency, the symbol digit, and all parts of the Stroop test (color, word, and interference). Rate of change (points/year) was calculated by subtracting cognitive score at the first assessment from the score at the last follow‐up assessment (or most complete data set) divided by the time between these assessments in years. The rate of change in motor decline was calculated using the total motor score from the UHDRS'99.

M, male; F, female; UHDRS, Unified Huntington's Disease Rating Scale.

### Genotyping

SNP genotyping was undertaken using predesigned assays (Applied Biosystems, Warrington, UK) tagging the R47H variant of *TREM2* (SNP: rs75932628) and 3 SNPs of the *TLR4* gene (SNP: rs1927911, rs1927914, rs10116253) and run on a Quantstudio 7 Flex Real‐Time PCR System (ThermoFisher, Waltham, MA, USA), according to the manufacturer's instructions. To validate the results, 192 DNA samples randomly selected were regenotyped 3 times in triplicate without any inconsistencies observed among those samples.

### Tissue Microarray Preparation

The Cambridge Brain Bank provided anonymous paraffin‐embedded tissue blocks from HD patients (N = 16 [n = 5 grade 3; n = 11 grade 4]) and age‐matched and sex‐matched controls (N = 9) known not to have any neurological or psychiatric disorders (Table 2). Striatal tissue was available for all cases. Demographic data were obtained from the Brain Bank. The pathological severity of HD was scored according to the Vonsattel grading system.^41^

**Table 2:** Demographic Details of the Postmortem Brain Sample Cases

HD Cases	Grade	Age	Sex
H614	3	42	F
H659	3	43	F
H679	3	51	M
H700	3	57	M
H709	3	79	F
H665	4	70	F
H669	4	53	M
H671	4	72	F
H682	4	40	M
H692	4	43	F
H693	4	26	F
H707	4	39	M
H710	4	43	M
H718	4	65	F
H720	4	68	M
H725	4	58	M
Mean ± SD		53.1 ± 14.7	
Ratio F:M			8:8

Controls	Age	Sex
C568	69	M
NP16.28	68	M
NP16.59	60	F
PT88	72	M
PT129	50	F
PT149	56	M
PT151	76	M
PT155	39	F
PT172	45	F
Mean ± SD	59.4 ± 12.9	
Ratio F:M		4:5

HD, Huntington's disease; SD, standard deviation; F, female; M, male.

After a preliminary hematoxylin and eosin and luxol fast blue staining, all of the blocks were assessed by a neuropathologist to mark the putamen and caudate and sent to the Integrated Systems Engineering (ISENET, Milan, Italy) for TMA assembly.^42^ A semiautomated tissue array device (Galileo TMA CK4500 platform, ISENET, Milan, Italy) with a needle punch of diameter 0.2 mm was inserted into the marked areas of the donor block within the putamen. Different donor tissue cores were inserted into precored holes in a recipient paraffin wax block according to the array coordinates defined in the predetermined template.

### Immunohistochemistry and Quantification

Immunohistochemistry was performed on 10‐μm thick sections from the TMAs (or single‐section slides in the case of cerebral cortex) using TREM2 and TLR4 antibodies and following standard protocols. Deparaffinized and rehydrated tissue sections were incubated overnight at 4°C with the following primary antibodies: mouse monoclonal anti‐TREM2 (1:200; Abcam, Toronto, Canada) and mouse monoclonal anti‐TLR4 (1:100; Abcam, Toronto, Canada). The labeling was revealed with the ABC Elite Vectastain Kit (Vector Laboratories, Peterborough, UK). The sections were then incubated for 2 hours at room temperature with the biotinylated secondary antibody (1:500; Vector Laboratories, Peterborough, UK) and, following washes in phosphate‐buffered saline, horseradish peroxidase Avidin‐D (Vector Laboratories, Peterborough, UK) was added for 1 hour at room temperature and visualized with 3‐3'diaminobenzidine as the chromogen. Controls included staining after omitting the primary antibody and were consistently negative for any staining.

Individual immunolabeled TMA sections were scanned on a Leica Aperio AT2 (Leica Biosystems, Buffalo Grove, IL, USA) at 20× magnification with a resolution of 0.5‐μm per pixel and visualized on ImageScope v12.4.0.7018 (Leica Biosystems, Buffalo Grove, IL, USA). Quantification was performed blinded to case identity. Both a positive cell detection and optical density analyses were performed using QuPath software^56^ (version 0.1.2). For analysis of the number of cells expressing each marker and the relative optical density in the cortex, 10 images of a 20× field of view per section were taken using a E600 epifluorescence microscope equipped with a DMX1200 digital camera driven by the Automatic Camera Tamer software (Nikon, Melville, NY, USA), and staining was analyzed using the Fiji image analysis software.^57^ The average value of all images per case was used for statistical analysis.

### Statistical Analysis

A χ^2^ test was used to compare the allele frequency of each variant with that expected for a population in Hardy‐Weinberg equilibrium. Fisher's exact test was used to compare the distribution of genotypes. Only genotyped individuals for whom a complete data set was available for at least 2 visits, a minimum of 1 year apart, were included in the analysis,^43^, ^44^ as we have done previously in this cohort.^15^ Baseline demographic and clinical data were compared between groups using 2‐tailed *t* tests (2 groups) and analysis of Aariance (more than 2 groups). We first assessed the annual change in cognitive performance based on a composite cognitive score, a sum of individual scores on the verbal fluency and the symbol digit tests as well as all parts of the Stroop test (color, word, and interference). Rate of change (points/year) was calculated by subtracting the cognitive score at the first assessment from the score at the last follow‐up assessment (or most complete data set) divided by the time between these assessments in years, as we have done previously.^15^, ^43^, ^44^ We then measured the rate of change in motor decline, calculated using the total motor score from the UHDRS'99 collected at the same visits as described previously using an equivalent formula. Outliers were identified and the data were winsorized using Tukey's Hinge estimates. The Shapiro‐Wilk test was used to assess the distribution of variables (motor, functional, and cognitive scores). Where data were not normally distributed, a Mann‐Whitney *U* test was used. A *P* value <0.05 was defined as statistically significant. Graphs were generated using GraphPad Prism (version 6.04 for Windows; GraphPad, San Diego, CA).

## Results

**Figure 1:**
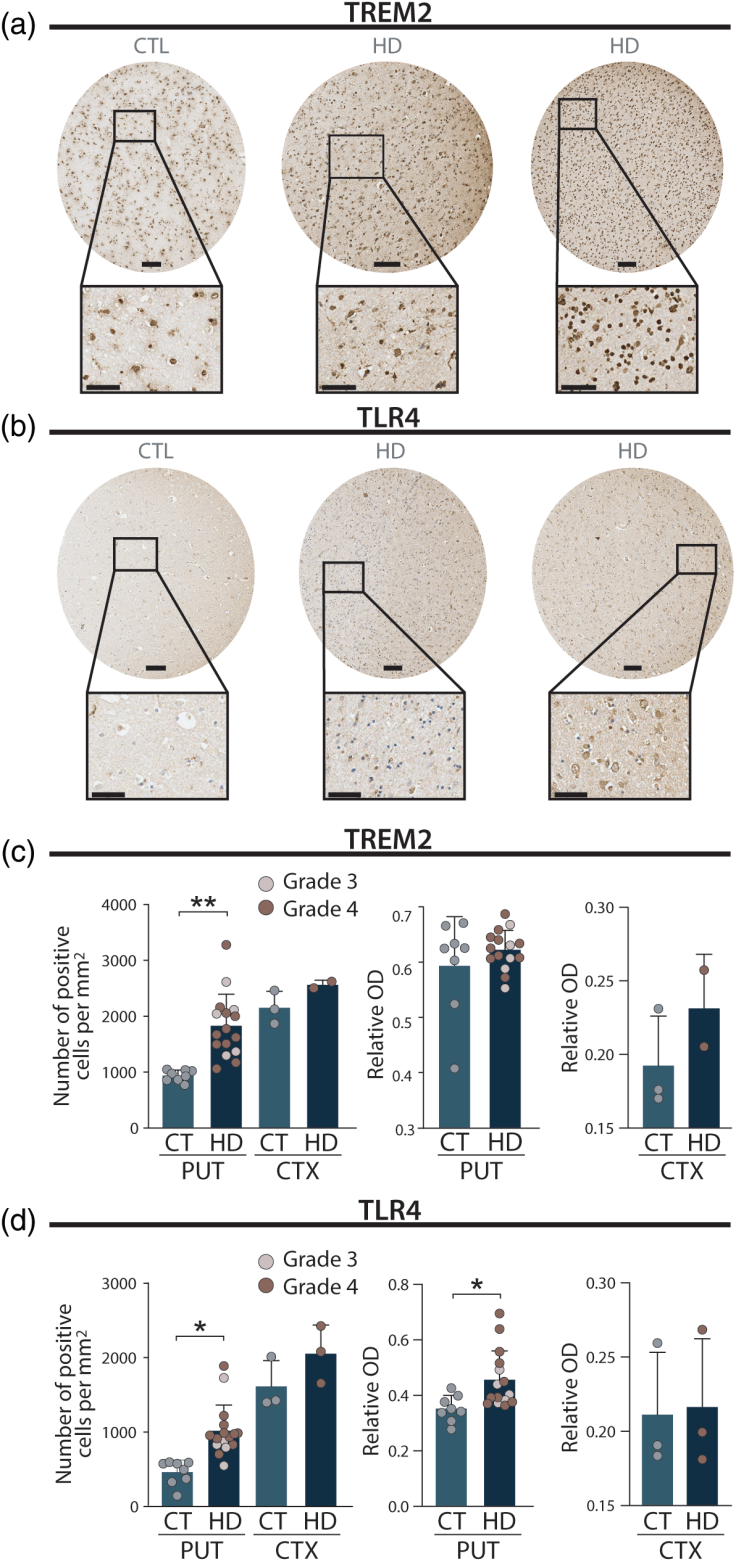
Increased expression of TLR4 and TREM2 in the striatum of HD patients. (**a**) Representative images of TREM2 immunostaining of putamenal tissue punches from tissue microarrays from HD brains of pathological grades 3 and 4 as well as a control brain. Scale bars = 100 μm in punch, 50 μm in inset. (**b**) Representative images of TLR4 immunostaining of putamenal tissue punches from tissue microarrays from HD brains of grades 3 and 4 as well as a control brain. Scale bars = 100 μm in punch, 50 μm in inset. (**c**) Quantification of the number of TREM2‐positive cells per mm^2^ in control and HD brains. Student's *t* test: ***P < 0.001, as compared to the control group. (**d**) Quantification of the number of TLR4‐positive cells per mm^2^ in control and HD brains. Student's *t* test: ***P < 0.001, as compared to the control group. CT/CTL, Controls; CTX, Cortex; HD, Huntington's Disease; PUT, Putamen; TLR4, toll‐like receptor 4; TREM2, triggering receptor expressed on myeloid cells 2. [Color figure can be viewed at http://wileyonlinelibrary.com]

To investigate the potential contribution of TREM2 and TLR4 to HD, we first assessed the protein expression levels in the striatum of HD patients using postmortem tissue. To make this analysis as consistent as possible across different specimens, we built TMAs with striatal tissue from HD patients and controls (Fig. 1a,b). Quantification of the number of cells expressing TREM2 and TLR4 revealed a significant increase in both markers in HD patients when compared with controls (Fig. 1c,d; *P* < 0.001). Moreover, there was also an increase in relative optical density of TLR4 in the striatum of HD patients when compared with controls (Fig. 1d; *P* < 0.05). We also sought to look at the expression of these 2 markers in the cortex of a subgroup of patients and controls. Although the number of cases is too low to draw clear conclusions, we did not observe major differences in TREM2 and TLR4 labeling in the cortex between HD cases and controls (Fig. 1c,d).

We next sought to determine whether *TREM2* and *TLR4* gene polymorphisms had any impact on disease progression and clinical expression using genotype–phenotype analysis. We thus genotyped 830 HD patients from the EHDN for the *TREM2* R47H variant (rs75932628) and 3 *TLR4* SNPs (rs1927911, rs1927914, rs10116253) (Table 3). Patients were divided into 2 main groups based on the allelic frequencies as described previously for other genetic variants^15^, ^43^, ^44^; those that were homozygous for the rare allele were combined with heterozygous cases as summarized in Table 3. Complete clinical data for 2 independent assessments at least a year apart were available for all 830 individuals who were then included in the analysis. Although we found no association between AoO and motor and cognitive declines, nor between CAG repeats length and motor and cognitive declines, the already established negative correlation between AoO and CAG repeat length was reproduced in the total population (Kendall's tau_b_ ‐0.255, *P* < 0.0001; Supporting Information Fig. 1).

**Table 3:** SNP Analysis

Gene	SNP	Genotype	N	Motor*	*P*	N	Functional*	*P*	N	Cognitive*	*P*
TLR4	rs1927914 (G/A)	A/A	337	5.15 (6.56)	0.039a	337	−0.92 (1.53)	0.828	323	−11.50 (24.88)	0.686
		G carriers	473	3.89 (7.05)		473	−0.85 (1.32)		212	−12.04 (24.61)	
TLR4	rs1927911 (A/G)	G/G	431	4.94 (7.07)	0.05a	431	0.90 (1.50)		276	−12.18 (26.98)	0.828
		A carriers	379	3.82 (6.91)		380	−0.85 (1.31)	0.511	257	−11.62 (22.05)	
TLR4	rs10116253 (T/C)	T/T	434	4.80 (6.79)	0.087	434	−0.90 (1.49)	0.604	281	−11.96 (25.29)	0.547
		C carriers	377	3.92 (7.21)		377	−0.86 (1.31)		255	−11.52 (23.57)	
TREM2	rs75932628 (H47R ‐ C/T)	C/C	817	4.47 (7.01)	0.914	817	−0.87 (1.40)	0.138	539	−11.74 (24.88)	0.018a
		T carriers	13	4.69 (11.79)		13	−1.25 (2.02)		9	−28.36 (26.47)	

* Median change (standard error of the ratio change of points/years).

SNP, single nucleotide polymorphism.

**Figure 2:**
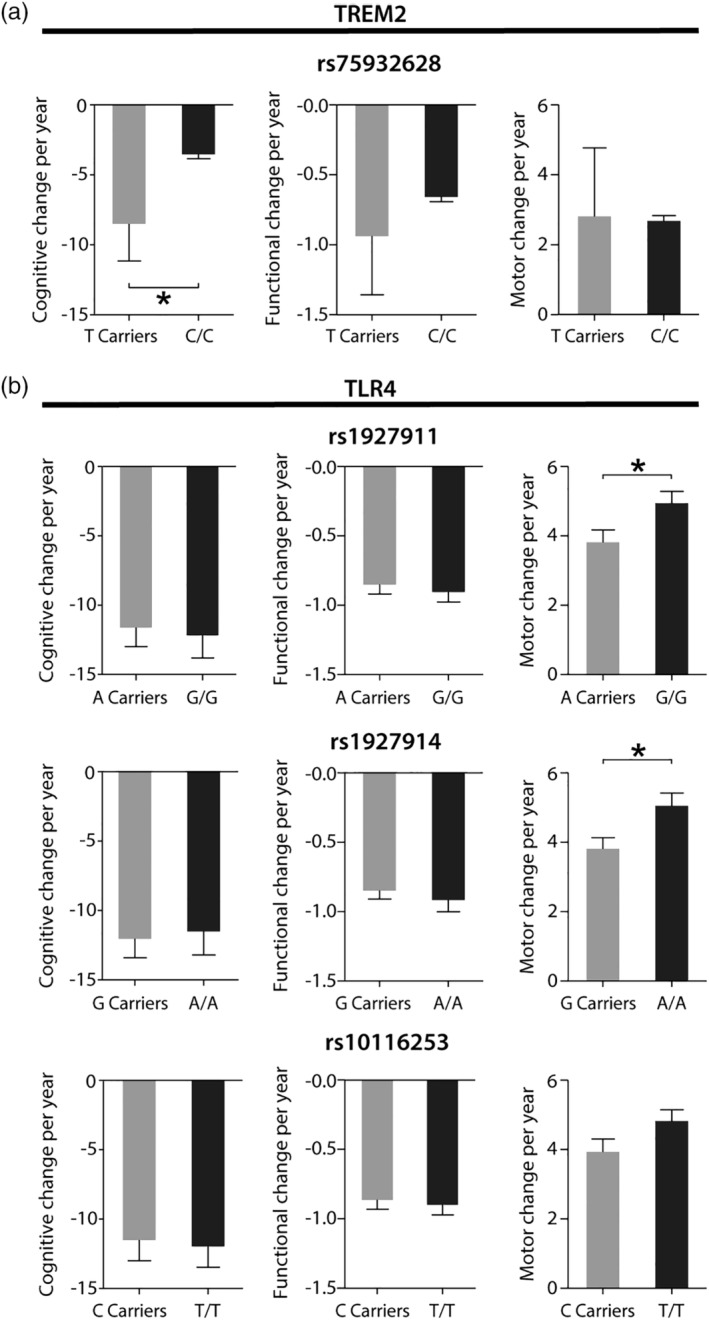
The effect of TREM2 and TLR4 single nucleotide polymorphisms variants on motor and cognitive decline in HD. (**a**) Graph showing a more severe cognitive decline in T carriers of the rs75932628 single nucleotide polymorphisms variant. Distribution was compared using Mann‐Whitney *U* test, *P < 0.05. (**b**) Graph showing a more severe motor decline in G/G carriers of the rs1927911 polymorphism as well as in A/A carriers of the rs1927914 polymorphism. Distribution was compared using Mann‐Whitney *U* test, *P < 0.05. TLR4, toll‐like receptor 4; TREM2, triggering receptor expressed on myeloid cells 2.

Nonparametric comparison showed that there was a significantly higher rate of cognitive decline in *TREM2* rs1927911 T carriers when compared with C/C patients, whereas this SNP did not impact on annual changes in functional capacity nor motor change (Fig. 2a; *P* < 0.05 and Table 3). However, overall motor decline per year was significantly higher in *TLR4* rs1927911 G/G when compared with A carriers as well as in *TLR4* rs1927914 A/A patients when compared with G carriers (Fig. 2b; *P* < 0.05 and Table 3). No changes in cognitive nor functional decline were associated with these 2 SNPs. Furthermore, when comparing *TLR4* rs10116253 T/T with C carriers, no association with any of the 3 clinical assessments could be detected (Fig. 2b and Table 3). Taken together, these results suggest that *TREM2* and *TLR4* may be genetic modifiers for disease progression but play different roles in contributing to the clinical phenotypes.

## Discussion

This is the first genotype–phenotype study assessing the influence of genes related to inflammation in the progression of HD. We first showed that there is an increased number of cells expressing TREM2 and TLR4 in the striatum of the HD brain, although only TLR4 showed increased protein expression at this site. We then sought to investigate the clinical significance of this by looking into the impact of common variants in these genes on clinical progression in a large cohort of patients with HD. We found that *TREM2* rs1927911 was associated with the rate of cognitive decline, whereas *TLR4* rs1927911 and *TLR4* rs1927914 were both associated with the rate of motor decline. Although we found no association between AoO and motor and cognitive declines, nor between CAG repeat length and motor and cognitive declines, the established negative correlation between AoO and CAG repeat length was reproduced in our population. Furthermore, there was no association between the SNPs *TREM2* rs1927911 and TLR4 rs1927911 and TLR4 rs1927914 and AoO and CAG repeat length. This implies that the influence of those genotypes on the rate of cognitive or motor decline is independent of AoO and CAG repeat length.

TLR4, a pattern recognition receptor, has also been associated with misfolded protein clearance. For instance, the uptake of α‐synuclein by microglia has been shown to depend at least in part on TLR4 in models of α‐synucleinopathies.^45^, ^46^ This receptor is also responsible for the α‐synuclein‐induced proinflammatory response in astrocytes^47^ and triggers the amyloid‐β‐induced activation of microglia in AD models.^48^ As such, it is not unexpected that TLR4 is also involved in the inflammatory response in HD. Consistent with this, the Nuclear Factor kappa‐light‐chain‐enhancer of activated B cells (NF‐κB) pathway, a key signaling cascade downstream of TLR4, has been shown to interact with mutant huntingtin protein exon 1 in mice.^49^ Moreover, a recent study reports that N171‐82Q HD mice lacking TLR4 have their lifespan significantly extended.^50^ The functional impact of the different *TLR4* polymorphisms on glial cells and neurons, which seems to impact on motor function rather than cognition, is however not known. Given that we found TLR4 expression to be increased specifically in the putamen of HD patients, this could mean that these SNPs are affecting the expression levels or the affinity of the receptor to the adaptor proteins, thus impacting on motor functions. Nonetheless, TLR4‐deficient mice have been reported to have impaired motor functions, a feature that was attributed to TLR4 neuronal expression in the cerebellum, although the striatum was not assessed pathologically and no cognitive tasks were performed.^51^

Consistent with the absence of an increase in TREM2 relative optical density in the striatum of HD cases, we found no difference in motor progression in patients carrying the *TREM2* R47H variant. The potential role of *TREM2* variants as a factor linked to cognitive progression of HD supports the hypothesis that inflammation might also contribute to the cognitive impairments seen in this disorder. TREM2 attenuates macrophage activation^52^ and microglia expressing the R47H variant have been reported to have a reduced capacity to bind to phospholipids in an AD model, suggesting that TREM2 senses changes in the lipid microenvironment that result from Aβ accumulation and neuronal degeneration, which triggers signals that activate microglial capacity to limit Aβ accumulation.^53^ As such, similar mechanisms related to the triggered activation of microglia by mutant huntingtin protein could underlie the more severe cognitive decline in patients with the R47H variant. Interestingly, a recent study reports more hyperphosphorylated tau in the cortex of an AD mouse model carrying the human *TREM2* R47H variant, which was thought to result from a reduction of microgliosis around amyloid‐β plaques. This in turn could facilitate the local seeding and spreading of tau,^54^ findings which were also reported in the AD patient brain where a reduced microglial accumulation around plaques associated with higher pathological tau burden was found in *TREM2* R47H HD patients.^55^ However, it still remains unclear as to why TREM2 seems to influence only cognition and not motor decline in our HD study, but it could relate to an effect it may have on the distribution of pathology. Although the postmortem samples available for our study does not allow one to conclude on TREM2 cortical expression levels in HD nor to assess whether such a reduction of microgliosis around mutant huntingtin protein inclusions exists in the cortex of *TREM2* R47H HD patient carriers, it will be interesting to look at whether this could underlie the link between the more aggressive cognitive decline suggested by our genotype–phenotype study in *TREM2* R47H HD patients.

Although our results implicate TLR4 and TREM2 in the clinical progression of HD, our study has a number of limitations. First, in this study, cognitive data from only N = 9 patients carrying the rs75932628 (H47R ‐ C/T) genotype were available, and thus the interpretation of these results should be done with great caution. Further studies with a larger number of patients carrying that genotype are now needed. Second, for some patients, AoO was defined by retrospective interviews of patients and the patient's history, which can be unreliable. Third, the pathological analysis we performed is very limited in the number of samples and regions assessed, and thus more extensive analyses in larger numbers of brain areas and patients would be useful. In addition, although pathological observations can provide evidence as to whether a factor could be involved in disease pathogenesis, it does not demonstrate causality and as such further in vitro and animal studies will need to be done before conclusions on pathophysiological mechanisms can be made.

In summary, we have shown that TREM2 and TLR4 are linked to the clinical progression and pathology of HD and as such warrant further investigation, including whether therapies modulating these pathways could be useful in slowing down disease progression.

## Author Roles

1. Research Project: A. Conception, B. Organization, C. Execution; 2. Statistical Analysis: A. Design, B. Execution, C. Review and Critique; 3. Manuscript Preparation: A. Writing the First Draft, B. Review and Critique.

R.V.: 1A,1B, 1C, 2A, 2B, 2C, 3A, 3B

A.K.: 2A, 2C, 3B

E.M.L.: 1C, 2B, 3B

L.C.: 1C, 3B

K.S.A.: 1C, 2C, 3B

A.L.S.: 1C, 3B

I.B.: 1B, 3B

R.A.B.: 1B, 3B

J.D.‐O.: 1A, 1B, 2A, 2C, 3A, 3B

## Financial Disclosures of all authors (for the preceding 12 months)

The research leading to these results has received funding from the European Research Council under the European Union's Seventh Framework Programme: FP/2007‐2013 NeuroStemcellRepair (no. 602278) to R.A.B. and Fonds du Québec en Recherche, Santé to J.D.O. R.V. has received funding from Alzheimer's Research UK. A.K. has been supported by a PhD scholarship from the Onassis Foundation and a studentship by the Alborada Trust and is currently funded by a grant from the Evelyn Trust. R.A.B. has received grants from National Institute for Health Research( NIHR), Medical Research Council (MRC), Wellcome Trust, Parkinson's UK, Huntington's Disease Association, Rosetrees Trust, Cure Parkinson's Trust, royalties from Wiley and Springer‐Nature, and consultancy monies from Living Cell Technologies (LCT), F‐Prime, Fujifilm Cellular Dynamics International, and Oxford Biomedica. R.A.B. is an NIHR senior investigator. J.D.O. is receiving salary support from Fonds du Québec en Recherche, Santé and Parkinson Québec, and is receiving funding from the Canada Foundation for Innovation (CFI) and Parkinson Canada. The Cambridge Brain Bank is supported in part by NIHR.

## Supporting information


**Appendix S1**: Supporting Information

**Supplementary Figure 1 Correlation between CAG repeat length and AoO**. Longer CAG repeats lead to early disease onset. Overall, Kendall's taub ‐0.255, p < 0.0001.
