# Supplementary material for: Association Between Toll‐Like Receptor 4 (TLR4) and Triggering Receptor Expressed on Myeloid Cells 2 (TREM2) Genetic Variants and Clinical Progression of Huntington's Disease
Source: Mov Disord. 2019 Nov 14;35(3):401–8. doi: 10.1002/mds.27911 (PMC7154663; doi:10.1002/mds.27911)
Supplement: Supplementary file 1 — Appendix S1: Supporting Information [file MDS-35-401-s001.doc]

REGISTRY 2004-2010

**Registry Steering committee:** A-C Bachoud-Lévi, AR Bentivoglio, I Biunno, RM Bonelli, J-M Burgunder, SB Dunnett, JJ Ferreira, OJ Handley, A Heiberg, T Illmann, GB Landwehrmeyer, J Levey, Maria Dolores Martinez-Jaurrieta

JE Nielsen, S Pro Koivisto, M Päivärinta, RAC Roos, A Rojo Sebastián, SJ Tabrizi, W Vandenberghe, C Verellen-Dumoulin, J Zaremba, T Uhrova, J Wahlström

**Language coordinators:** Katrin Barth, Monica Bascuñana Garde, Sabrina Betz, Reineke Bos, Leonor Correia-Guedes, Daniel Ecker, Ana Maria Finisterra, Olivia J Handley, Christine Held, Matilde Laurà, Asunción Martínez Descals, Tiago Mestre, Sara Minster, Daniela Monza, Michael Orth, Helene Padieu, Nadia Peppa, Susana Pro Koivisto, Amandine Rialland, Niini Røren (formerly Heinonen) Pavla Šašinková, Jenny Townhill,  Patricia Trigo Cubillo, Marleen R van Walsem, Marie-Noelle Witjes-Ané, Daniel Zielonka, Eugeniusz Zielonka, Paola Zinzi

**AUSTRIA**

**Graz (LKH Graz, Abteilung für Psychiatrie):** Raphael M. Bonelli, Brigitte Herranhof, Karen Hecht, Anna Holl (formerly Hödl), Hans-Peter Kapfhammer, Michael Koppitz, Sabine Lilek, Markus Magnet, Nicole Müller, Daniela Otti, Annamaria Painold, Karin Reisinger, Monika Scheibl, Helmut Schöggl, Jasmin Ullah

**BELGIUM**

**Charleroi (Institut de Pathologie et de Génétique (IPG)):** Pascale Ribaï, Christine Verellen-Dumoulin

**Leuven:** (Universitair Ziekenhuis Gasthuisberg,): Andrea Boogaerts, Wim Vandenberghe, Dimphna van Reijen

**CZECH REPUBLIC**

**Prague (Extrapyramidové centrum, Neurologická klinika, 1. LF UK a VFN):** Jiří Klempíř, Veronika Majerová, Jan Roth, Irena Stárková

**DENMARK**

**Copenhagen University Hospital (Rigshospitalet, Memory clinic):** Lena Hjermind, Oda Jacobsen, Jørgen Nielsen, Jette Stockholm, Ida Unmack Larsen, Tua Vinthev-Jensen

**FINLAND**

**Turku-Suvituuli (Rehabilitation Centre Suvituuli):** Heli Hiivola, Kirsti Martikainen, Katri Tuuha

**GERMANY**

**Aachen (Universitätsklinikum Aachen, Neurologische Klinik):** Christoph Michael Kosinski, Eva Milkereit, Daniela Probst, Christian Sass, Johannes Schiefer, Christiane Schlangen, Cornelius J. Werner

**Bochum (Huntington-Zentrum (NRW) Bochum im St. Josef-Hospital):** Jürgen Andrich, Gisa Ellrichmann, Rainer Hoffmann, Barbara Kaminski, Peter Kotz, Christian Prehn, Carsten Saft

**Dinslaken (Reha Zentrum in Dinslaken im Gesundheitszentrums Lang):** Herwig Lange, Robert Maiwald

**Dresden (Universitätsklinikum Carl Gustav Carus an der Technischen Universität Dresden, Klinik und Poliklinik für Neurologie):** Matthias Löhle, Simone Schmidt, Alexander Storch, Annett Wolz, Martin Wolz

**Freiburg (Universitätsklinik Freiburg, Neurologie):** Philipp Capetian, Johann Lambeck, Birgit Zucker

**Hamburg (Universitätsklinikum Hamburg-Eppendorf, Klinik und Poliklinik für Neurologie):** Kai Boelmans, Christos Ganos, Ute Hidding, Jan Lewerenz, Alexander Münchau, Michael Orth, Jenny Schmalfeld, Lars Stubbe, Simone Zittel

**Heiligenhafen (Psychatrium Heiligenhafen**): Walburgis Heinicke

**Marburg KPP (Klinik für Psychiatrie und Psychotherapie Marburg-Süd):** Bernhard Longinus

**München (Huntington-Ambulanz im Neuro-Kopfzentrum - Klinikum rechts der Isar der Neurologischen Klinik und Poliklinik der Technischen Universität München):** Antje Lüsebrink, Mark Mühlau, Alexander Peinemann, Michael Städtler, Adolf Weindl, Juliane Winkelmann, Cornelia Ziegler

**Münster (Universitätsklinikum Münster, Klinik und Poliklinik für Neurologie):** Natalie Bechtel, Heike Beckmann, Stefan Bohlen, Eva Hölzner, Herwig Lange, Ralf Reilmann, Stefanie Rohm, Silke Rumpf , Sigrun Schepers

**Taufkirchen (Isar-Amper-Klinikum - Klinik Taufkirchen (Vils)):** Antonie Beister, Matthias Dose, Kathrin Hammer, Janina Kieni, Gabriele Leythaeuser, Ralf Marquard, Tina Raab, Sven Richter, Amina Selimbegovic-Turkovic, Caroline Schrenk, Michele Schuierer, Alexandra Wiedemann

**Ulm (Universitätsklinikum Ulm, Neurologie):** Katrin Barth, Andrea Buck, Julia Connemann, Daniel Ecker, Carolin Geitner, Christine Held, Andrea Kesse, Bernhard Landwehrmeyer, Christina Lang, Jan Lewerenz, Franziska Lezius, Solveig Nepper, Anke Niess, Michael Orth, Ariane Schneider, Daniela Schwenk, Sigurd Süßmuth, Sonja Trautmann, Patrick Weydt

**ITALY**

**Bari Clinica Neurologica - Neurophysiopatology of Pain Unit UNIVERSITA' DI BARI):** Claudia Cormio, Vittorio Sciruicchio, Claudia Serpino, Marina de Tommaso

**Florence (Dipartimento di Scienze Neurologiche e Psichiatriche Universita' degli Studi di Firenze-Azienda Ospedaliera Universitaria Careggi):** Elisabetta Bertini, Elena Ghelli, Andrea Ginestroni, Francesca Massaro, Claudia Mechi, Marco Paganini, Silvia Piacentini, Silvia Pradella, Anna Maria Romoli, Sandro Sorbi

**Genoa (Dipartimento di Neuroscienze, Oftalmologia e Genetica (DiNOG)
Università di Genova):** Giovanni Abbruzzese, Monica Bandettini di Poggio, Emilio Di Maria, Giovanna Ferrandes, Paola Mandich, Roberta Marchese

**Milan (Fondazione IRCCS Istituto Neurologico Carlo Besta**): Alberto Albanese, Daniela Di Bella, Stefano Di Donato, Cinzia Gellera, Silvia Genitrini, Caterina Mariotti, Daniela Monza, Lorenzo Nanetti, Dominga Paridi, Paola Soliveri, Chiara Tomasello

**Naples (Dipartimento di Neuroscienze, Scienze Riproduttive e Odontostomatologiche, Università Federico II):** Giuseppe De Michele, Luigi Di Maio, Carlo Rinaldi, Pierpaolo Sorrentino, Cinzia Valeria Russo, Elena Salvatore, Tecla Tucci

**Pozzilli (IS) (Centro di Neurogenetica e Malattie Rare – IRCCS Neuromed):** Enrico Amico, Milena Calenna, Alba Di Pardo, Mariagrazia Favellato, Vittorio Maglione, Irene Mazzante, Martina Petrollini, Ferdinando Squitieri

**Rome (Istituto di Neurobiologia e Medicina Molecolare & Istituto di Scienze e Tecnologie della Cognizione /CNR, Istituto di Neurologia Università Cattolica del Sacro Cuore):** Marina Frontali, Arianna Guidubaldi, Tamara Ialongo, Gioia Jacopini, Carla Piano, Anna Rita Bentivoglio, Silvia Romano, Francesco Soleti, Maria Spadaro, Paola Zinzi

**NETHERLANDS**

**Enschede (Medisch Spectrum Twente):** Monique S.E. van Hout, Jeroen P.P. van Vugt, A. Marit de Weert

**Groningen (Polikliniek Neurologie**): J.J.W. Bolwijn, M. Dekker, K.L. Leenders, J.C.H. van Oostrom

**Leiden (Leiden University Medical Centre (LUMC**)): Reineke Bos, Eve M. Dumas, Simon J. A. van den Bogaard, Raymund A.C. Roos, Ellen P. ‘t Hart,

**Nijmegen (Universitair Medisch Centrum St. Radboud, Neurology**): Berry Kremer, C.C.P. Verstappen

**NORWAY**

**Oslo University Hospital (Rikshospitalet, Dept. of Medical Genetics and Dep. of Neurology):** Olaf Aaserud, Jan Frich, Arvid Heiberg, Marleen R van Walsem, Raghild Wehus

**Oslo University Hospital (Ulleval**, **Dept of Medical Genetics and Department)**: Kathrine Bjørgo, Madelein Fannemel, Per Gørvell, Eirin Lorentzen, Susana Pro Koivisto, Lars Retterstøl, Bodil Stokke

**Trondheim (St. Olavs Hospital):** Inga Bjørnevoll, Sigrid Botne Sando

**POLAND**

**Gdansk (St. Adalbert Hospital, Gdansk, Medical University of Gdansk, Neurological and Psychiatric Nursing Dpt.):** Artur Dziadkowiak, Emilia Sitek, Jaroslaw Slawek, Witold Soltan

**Katowice (Medical University of Silesia, Katowice):** Magdalena Błaszczyk, Magdalena Boczarska-Jedynak, Ewelina Ciach-Wysocka, Barbara Jasinska-Myga, Gabriela Kłodowska – Duda, Gregorz Opala, Daniel Stompel, Agnieszka Gorzkowska

**Krakow (Krakowska Akademia Neurologii):** Krzysztof Banaszkiewicz, Dorota Boćwińska, Kamila Bojakowska-Jaremek, Małgorzata Dec, Malgorzata Krawczyk, Monika Rudzińska, Elżbieta Szczygieł, Andrzej Szczudlik, Magdalena Wójcik, Anna Wasielewska

**Poznan (Poznan University of Medical Sciences, Poland):** Anna Bryl, Anna Ciesielska, Aneta Klimberg, Jerzy Marcinkowski, Husam Samara, Justyna Sempołowicz, Daniel Zielonka

**Warsaw-MU (Medical University of Warsaw, Neurology):** Anna Gogol (formerly Kalbarczyk), Zygmunt Jamrozik, Piotr Janik, Hubert Kwiecinski

**Warsaw-IPiN (Institute of Psychiatry and Neurology Dep. of Genetics, First Dep. of Neurology):** Jakub Antczak, Wioletta Krysa, Katarzyna Jachinska, Maryla Rakowicz, Danuta Ryglewicz, Iwona Stępniak, Anna Sułek, Grzegorz Witkowski, Jacek Zaremba, Elzbieta Zdzienicka, Karolina Ziora-Jakutowicz

**PORTUGAL**

**Lisbon- (Hospital de Santa Maria, Neurological Clinical Research Unit, Instituto de Medicina Molecular):** Miguel Coelho, Joaquim J Ferreira, Leonor Guedes, Tiago Mendes, Tiago Mestre, Anabela Valadas

**SPAIN**

**Barcelona-Bellvitge (Hospital Universitari de Bellvitge):** Jordi Bas, Núria Busquets

Matilde Calopa

**Barcelona-Merced (Hospital Mare de Deu de La Merced):** Marina Dalmau Elorza, Cristóbal Díez-Aja López, Santiago Durán-Sindreu Terol, Misericordia Floriach Robert, Belén Garzón Ruíz, Ana González Casado, Isabel Haro Martínez, Celia Mareca Viladrich, Regina Pons i Càrdenas, Elvira Roca, Joan Roig Llesoy, Jesús Miguel Ruiz Idiago, Mar Ruíz Vergara, Socorro Soriano García, Antonio Villa Riballo

**Barcelona-Hospital Mútua de Terrassa:** Miquel Aguilar Barbera, Dolors Badenes Guia, Laura Casas Hernanz , Judit López Catena, Ana Rojo Sebastián, Pilar Quiléz Ferrer, Gemma Tome Carruesco

**Burgos (Servicio de Neurología Hospital General Yagüe):** Esther Cubo, Natividad Mariscal Perez

**Granada (Hospital Universitario San Cecilio, Neurología**): Francisco Barrero, Blas Morales

**Madrid-Clinico (Hospital Clínico Universitario San Carlos):** María Fenollar, Rocío García-Ramos García, Purificacion Pin Quiroga, Susana Vázquez Rivera, Clara Villanueva

**Madrid RYC (Hospital Ramón y Cajal, Neurología):** Javier Alegre, Mónica Bascuñana, Juan Garcia Caldentey, Marta Fatás Ventura, Guillermo García Ribas, Justo García de Yébenes, José Luis López – Sendón Moreno, Patricia Trigo Cubillo

**Madrid FJD (Madrid-Fundación Jiménez Díaz):** Javier Alegre, Fernando Alonso Frech, Justo García de Yébenes, Rosa Guerrero, Pedro J García Ruíz, Asunción Martínez-Descals, María José Saiz Artiga, Vicenta Sánchez

**Sevilla (Hospital Virgen Macarena):** Jose Manuel Garcia Moreno, Caroline Mendez Lucena, Fatima Damas Hermoso, Eva Pacheco Cortegana

**Palma (Hospital Son Dureta):** Aranzazú Gorospe, Inés Legarda, Penelope Navas Arques, María José Torres Rodríguez, Barbara Vives

**SWEDEN**

**Göteborg (Sahlgrenska University Hospital**): Radu Constantinescu, Gunnel Fredlund, Ulrika Høsterey-Ugander, Liselotte Neleborn-Lingefjärd , Jan Wahlström

**SWITZERLAND**

**Bern:** Jean-Marc Burgunder, Yanik Stebler **(Neurologische Klinik des Inselspitals),** , Alain Kaelin, Irene Romero, Michael Schüpbach, Sabine Weber Zaugg **(Zentrum für Bewegungsstörungen, Neurologische Klinik und Poliklinik)**

**U.K.**

**Aberdeen (NHS Grampian Clinical Genetics Centre & University of Aberdeen):**

Roisin Jack, Kirsty Matheson, Zosia Miedzybrodzka, Daniela Rae, Sheila Simpson, Fiona Summers, Alexandra Ure, Vivien Vaughan

**Birmingham (The Barberry Centre, Dept of Psychiatry**): Shahbana Akhtar, Jenny Crooks, Adrienne Curtis, Jenny de Souza (Keylock), Hugh Rickards, Jan Wright

**Cambridge (Cambridge Centre for Brain Repair, Forvie Site**): Matthew Armstrong, Roger A. Barker, Anna Di Pietro, Kate Fisher, Anna Goodman, Susan Hill, Deidre O’Keefe, Ann Kershaw, Sarah Mason, Nicole Paterson, Lucy Raymond, Rachel Swain

**Cardiff (Schools of Medicine and Biosciences, Cardiff University**): Jonathan Bisson, Monica Busse, Cynthia Butcher, Rebecca Cousins, Stephen Dunnett, Catherine Clenaghan, Sarah Hunt, Lesley Jones, Una Jones, Hanan Khalil, Sara Minster,  Michael Owen, Kathleen Price, Anne Rosser, Jenny Townhill

**Edinburgh (Molecular Medicine Centre, Western General Hospital, Department of Clinical Genetics):** Maureen Edwards, Teresa Hughes (Scottish Huntington´s Association), Marie McGill, Pauline Pearson, Mary Porteous, Paul Smith (Scottish Huntington´s Association), Adam Zeman

**Gloucester (Department of Neurology Gloucestershire Royal Hospital):** Liz Burrows, Amy Fletcher, Alison Harding, Fiona Laver, Mark Silva, Aileen Thomson

**Leeds (Chapel Allerton Hospital, Department of Clinical Genetics):** Kathy Barnes, Carol Chu, Emma Hobson, Stuart Jamieson, Ivana Markova, Jenny Thomson, Jean Toscano, Sue Wild, Pam Yardumian

**Fife (Scottish Huntington's Association Whyteman's Brae Hospital):** Peter Brockie, Jillian Foster, Nicola Johns, Sue McKenzie, Jean Rothery, Gareth Thomas, Shona Yates

**Leicester (Leicestershire Partnership Trust, Mill Lodge):** Colin Bourne, Jackie Clapton, Carole Clayton, Heather Dipple, Janet Grant, Diana Gross, Caroline Hallam, Julia Middleton, Ann Murch, Dawn Patino

**London (Guy's Hospital):** Thomasin Andrews, Andrew Dougherty, Fred Kavalier, Charlotte Golding, Hana Laing, Alison Lashwood, Dene Robertson, Deborah Ruddy, Anna Whaite

**London (The National Hospital for Neurology and Neurosurgery**): Thomasin Andrews, Stefania Bruno, Karen Doherty, Charlotte Golding, Nayana Lahiri, Marianne Novak, Aakta Patel, Elisabeth Rosser, Sarah Tabrizi, Rachel Taylor, Thomas Warner, Edward Wild

**Manchester (Genetic Medicine, University of Manchester, Manchester Academic Health Sciences Centre and Central Manchester University Hospitals NHS Foundation Trust):** Natalie Arran, Judith Bek, Jenny Callaghan, David Craufurd, Ruth Fullam, Marianne Hare, Liz Howard, Susan Huson, Liz Johnson, Mary Jones, Julie Snowden, Andrea Sollom, Jennifer Thompson, Helen Murphy, Emma Oughton, Lucy Partington-Jones, Dawn Rogers, Cheryl Stopford, Iris Trender-Gerhard, Nichola Verstraelen (formerly Ritchie), Leann Westmoreland

**Oxford (Oxford University Hospitals NHS Trust, Dept. of Neurosciences, University of Oxford):** Richard Armstrong, Kathryn Dixon, Andrea H Nemeth, Gill Siuda

**Plymouth (Plymouth Huntington Disease Service, Mount Gould Hospital):** David Harrison, Max Hughes, Andrew Parkinson, Beverley Soltysiak

**Sheffield (The Royal Hallamshire Hospital– Sheffield Children’s Hospital):** Oliver Bandmann, Alyson Bradbury, Helen Fairtlough, Kay Fillingham, Isabella Foustanos, Paul Gill, Mbombe Kazoka, Kirsty O’Donovan, Nadia Peppa, Katherine Tidswell, Oliver Quarrell
