# Supplementary material for: Association Between Toll‐Like Receptor 4 (TLR4) and Triggering Receptor Expressed on Myeloid Cells 2 (TREM2) Genetic Variants and Clinical Progression of Huntington's Disease
Source: Mov Disord. 2019 Nov 14;35(3):401–8. doi: 10.1002/mds.27911 (PMC7154663; doi:10.1002/mds.27911)
Supplement: Supplementary file 2 — Supplementary Figure 1 Correlation between CAG repeat length and AoO. Longer CAG repeats lead to early disease onset. Overall, Kendall's taub ‐0.255, p < 0.0001. [file MDS-35-401-s002.docx]

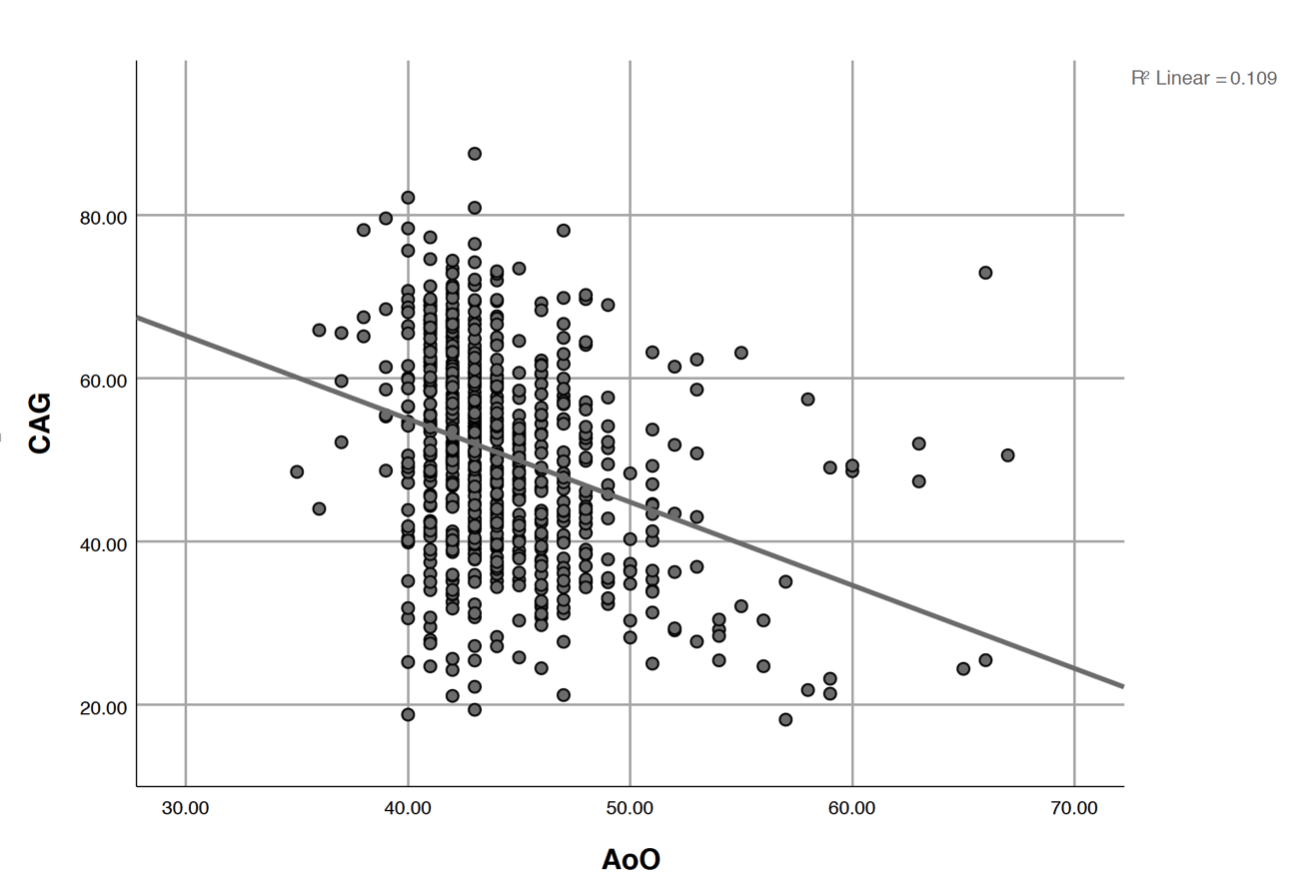


**Supplementary Figure 1**. **Correlation between CAG repeat length and AoO.** Longer CAG repeats lead to early disease onset. Overall, Kendall’s taub -0.255, p < 0.0001.
